# Effects of chlorogenic acid, epicatechin gallate, and quercetin on mucin expression and secretion in the Caco‐2/HT29‐MTX cell model

**DOI:** 10.1002/fsn3.818

**Published:** 2019-01-31

**Authors:** Tereza Volstatova, Alessandra Marchica, Zuzana Hroncova, Rodolfo Bernardi, Ivo Doskocil, Jaroslav Havlik

**Affiliations:** ^1^ Department of Microbiology, Nutrition and Dietetics Czech University of Life Sciences Prague Prague Czech Republic; ^2^ Department of Agricultural, Food and Agro‐Environmental Sciences University of Pisa Pisa Italy; ^3^ Department of Genetics and Breeding of Farm Animals Institute of Animal Science Prague Czech Republic; ^4^ Interdepartmental Research Center Nutrafood “Nutraceuticals and Food for Health” University of Pisa Pisa Italy; ^5^ Department of Quality of Agricultural Products Czech University of Life Sciences Prague Prague Czech Republic

**Keywords:** coculture of Caco‐2/HT29‐MTX cells, enzyme‐linked immunoassay, gene expression, phenolic constituents, RT‐PCR

## Abstract

Mucins are a family of large glycoproteins that represent the major structural components of the mucus and are encoded by 20 different mucin genes. Mucin expression can be modulated by different stimuli. In this study, we analyzed four mucins (MUC2, MUC3, MUC13, and MUC17) in coculture of Caco‐2/HT29‐MTX cells to demonstrate the variation in gene expression in the presence of antioxidant compounds like chlorogenic acid, epicatechin gallate, and quercetin (apple, tea, and coffee polyphenols, respectively). coculture of Caco‐2/HT29‐MTX cells was treated with polyphenols, and the expression of four mucins was determined by reverse‐transcriptase PCR. In addition, the secretion levels of MUC2 were established by enzyme‐linked immunoassay (ELISA) analysis. The results showed that each polyphenol compound induces different expression patterns of the mucin genes. Statistically significant up‐regulation of MUC17 was observed following incubation with epicatechin gallate and quercetin. ELISA results did not prove any significant differences in protein levels of MUC2 after treatment by the polyphenol compounds. The polyphenols considered in this study may influence mucin secretion and act on diverse salivary substrates to change the barrier properties of mucins for mucus secretion in different ways.

## 1 INTRODUCTION

The human gastrointestinal tract is covered with a mucus layer that acts as a physicochemical barrier to protect the underlying epithelium from pathogens and foreign antigens (Hews et al., 2017). The mucus layer consists of mucins, which are high molecular weight epithelial glycoproteins with a high content of clustered oligosaccharides (Byrd & Bresalier, 2004). The extensive, glycosylated tandem repeats that are characteristic of mucins protect the epithelium from inflammation and other forms of stress (Kufe, 2009). Mucins represent the main component of the intestinal mucus structure and enable its protective properties, and they are encoded by more than 20 different *MUC* genes (Boegh & Nielsen, 2015), which are divided into two different classes: transmembrane and secreted. Transmembrane mucins play important roles in preventing infection at mucosal surfaces, but also contribute to the development, progression, and metastasis of adenocarcinomas. They seem to have evolved to monitor and repair damaged epithelia, whereas this function can be “hijacked” by cancer cells (van Putten & Strijbis, 2017). Secreted mucins are either produced by mucosal cells that are present in the submucosal glands, or by specialized cells from apical surface epithelium, generally called Goblet cells (Tarang, Kumar, & Batra, 2012). Secreted mucins include MUC2, MUC5AC, MUC5B, MUC6, MUC7, MUC8, and MUC19, and the membrane‐bound mucins are MUC1, MUC3, MUC4, MUC12, MUC13, MUC14, MUC15, MUC16, MUC17, and MUC20 (Tarang et al., 2012). In the human intestine, MUC2 is the major secreted mucin of the mucosal layer (Hews et al., 2017). Mucins are characterized by a defined pattern of expression that can be modified by environmental factors and thereby involve an alteration of gene expression (Hollingsworth & Swanson, 2004). Recently, therapeutic approaches have focused on mucin regulation during inflammation and cancer in order to use mucins as therapeutic targets (Macha et al., 2015).

Previous studies demonstrated that dietary compounds, which interact with Goblet cells, could modify the secretion and composition of mucins. Some fibers, like sulfated polymers, and major short‐chain fatty acids present in the colon may increase mucin secretion (Barcelo et al., 2000; Deplancke & Gaskins, 2001; Sharma, Schumacher, Ronaasen, & Coates, 1995).

Polyphenols are the main class of plant secondary metabolites that show efficacy in the prevention of certain diseases, such as cancer, type II diabetes, and cardiovascular disease (Rothwell et al., 2013). They are characterized by the presence of several phenol rings, which are associated with generally complex structures of high molecular weight with one or more attached hydroxyl groups (Biasi et al., 2013). Recently, these polyphenols have gained considerable interest because of their potential health benefits; as such, they are likely the most studied class of molecules with nutritional interest in mind (Calani et al., 2012). Polyphenols are largely metabolized in tissues, such as the colon, small intestine, and liver, where they can exert several pharmacological effects, such as antioxidative and anticarcinogenic (Yang, Wang, Lu, & Picinich, 2009). The bioavailability of polyphenols in humans is abundantly discussed. The maximum concentration of parent compound in human plasma rarely exceeds 1 μM after the consumption of 10–100 mg of a single phenolic compound (Karakaya, 2004; Scalbert & Williamson, 2000). Following the ingestion of flavonoids as part of a normal diet, they undergo hydrolysis in the small intestine but are mostly poorly absorbed (Havlik & Edwards, 2018). After entering the proximal colon, they are often (but not always) transformed into simple phenolic compounds by the resident microbiota and may be absorbed for hepatic transformation and enter circulation (Havlik & Edwards, 2018; Selma, Espin, & Tomas‐Barberan, 2009). In the upper and lower digestive tract, epithelial cells are probably exposed to low, but physiologically relevant, concentrations of free polyphenols. It has been suggested that luminal concentrations of flavonoids, for example after consumption of 20 mg of quercetin‐rich food may peak at ~100 μM in the ceacum, and such low concentrations appear relevant for diet‐based studies (Havlik & Edwards, 2018).

Chlorogenic acid, epicatechin gallate, and quercetin were selected as representatives of the most abundant and well‐characterized dietary phenolics, since they occur naturally in apples, tea leaves, and coffee (Boyer & Liu, 2004; Calani et al., 2012; Yang et al., 2009).

In our study, we investigated how the gene expression of four mucins is affected by the presence of the three representative dietary plant polyphenols, in order to examine the effect of antioxidant compounds on mucin alteration in a coculture of intestine cancer cells: Caco‐2 and HT29‐MTX.

The four mucins in question were selected for several reasons. MUC2 is a major secreted mucin (Han, Deglaire, Sengupta, & Moughan, 2008), MUC3 is the most studied mucin of the adhering membrane class (Tarang et al., 2012), and MUC13 and MUC17 have only been recently discovered (Pelaseyed et al., 2014).

## 2 MATERIALS AND METHODS

### 2.1 Cell cultures and reagents

The Caco‐2 and HT29‐MTX cells lines are immortalized lines of heterogeneous human epithelial colorectal adenocarcinoma cells. The Caco‐2 cell line was purchased from the American Type Culture Collection (Rockville, Maryland, USA), and the HT29‐MTX cell line was obtained from Sigma‐Aldrich (Prague, Czech Republic). Both cell lines (passages P37 and P74, respectively) were grown in Dulbecco's Modified Eagle medium (DMEM) supplemented with 20% (v/v) fetal bovine serum, 1% nonessential amino acids, 100 U/ml Penicillin, and 100 μg/ml Streptomycin. All media and reagents were purchased from Sigma‐Aldrich (Saint‐Louis, USA). Cell cultures were incubated at 37°C in the presence of 5% (v/v) CO_2_, and the cell culture medium was replaced every 2 days. The cell lines were seeded in two NUNC 24‐well culture plates at concentrations of 3.6 × 10^4^ Caco‐2 cells and 0.4 × 10^4^ HT29‐MTX cells in 1 ml of DMEM for mucin expression and ribonucleic acid (RNA) studies. Cells were grown for 14 ± 1 days until 80% cell monolayer confluence. All culture plastic materials were purchased from Thermo Fisher Scientific (Waltham, Massachusetts, USA).

Chlorogenic acid, epicatechin gallate, and quercetin were selected as representatives of the most abundant and well‐characterized dietary phenolics, since they occur naturally in apples, tea leaves, and coffee. They were purchased from Extrasynthese (Genay Cedex, France). Stock solutions of the compounds in 80% ethanol were serially diluted in DMEM to a final concentration of 10 μM, keeping the ethanol concentration <1%.

### 2.2 MTT assay

Prior to the experiment, appropriate concentrations of the polyphenols were selected based on the results of cytotoxicity screenings (Biasi et al., 2013; Lee, Ji, & Sung, 2010). Cell viability was measured using the 3‐(4,5‐dimethylthiazolyl‐2)‐2,5‐diphenyltetrazolium bromide(MTT) assay. Caco‐2 cells, HT29‐MTX cells, and their cocultures were pre‐incubated in a NUNC 96‐well plate at a density of 2.5 × 10^3^ cells of Caco‐2 and HT29‐MTX cells/well and for coculture at a density of 3.6 × 10^3^ Caco‐2 cells and 0.4 × 10^3^ HT29‐MTX cells/well for 24 hr at 37°C in a 5% CO_2_ humidified atmosphere. Cells were treated with a twofold serial dilution of the compounds in the concentration range of 40–1,280 μmol/ml for 72 hr. Subsequently, MTT reagent (1 mg/ml) in DMEM was added to each well, and the plates were incubated for an additional 2 hr at 37°C. Culture supernatants were aspirated, and 100 μl of dimethylsulfoxide was added to each well. The absorbance was measured at 555 nm using the Tecan Infinite M200 reader (Tecan Austria GmbH, Grödig, Austria). The percentage of mortality in the presence of each concentration of extracts was plotted and used to determine the 50% inhibitory concentration (IC_50_).

### 2.3 Mucin expression and RNA assays

The old medium in a confluent 24‐well plate was aspirated, 1 ml of medium with 10‐μM concentration of the selected compound was added, and the plate was incubated for 48 hr. As a control, DMEM without the compound was added. All samples were tested in six replicates; three adjoining wells were used for RNA studies and three for mucin expression.

For RNA extraction and follow‐up reverse transcriptase PCR (RT‐PCR) analysis, the medium was aspirated after the treatment. Nonadhered cells were removed by washing the plate three times with sterile phosphate buffered saline (PBS), before treatment with 300 μl of 1% trypsin (Sigma‐Aldrich, Saint‐Louis, USA). Finally, the solution was suspended in PBS. To determine the amount of mucin secreted using ELISA, wells were released by scrubbing cells with pipette tips. The total contents of the wells were transferred into 15‐ml polypropylene tubes and centrifuged (2,000 × *g*, 10 min). Supernatants were kept at −18°C for later analysis.

### 2.4 RNA extraction and Real‐time RT‐PCR analysis

The total RNA from cells was extracted using RNeasy^®^ Mini Kit (Qiagen, Velno, Netherland), treated with Amplification Grade DNase I (Sigma‐Aldrich), and reverse‐transcribed into cDNA (400 ng per sample) using the iScript cDNA synthesis kit (Bio‐Rad, Hercules, CA, USA).

The products were stored at −20°C until analysis. β*‐Actin* was used as a reference gene after confirmation of its transcriptional stability in our experimental conditions. This reference gene was selected out of the three considered genes and amplified using specific primers for β*‐actin* (forward 5′‐CTTCCTGGGCATGGAGTC‐3′ and reverse 5′‐GCAATGATCTTGATCTTCATTGTG‐3′) (Johansson et al., 2008), and *GAPDH* (forward, 5′‐AGCCACATCGCTCAGACAC‐3′ and reverse, 5′‐GCCCAATACGACCAAATCC‐3′) (Tamagawa et al., 2012). The Hu18S primers were designed on the *Homo sapiens 18S rRNA* sequence (GenBank accession nos AJ844646) (forward, 5′‐TGGTGCATGGCCGTTCTT‐3′ and reverse, 5′‐AGCATGCCAGAGTCTCGTTCGT‐3′) (Martínez‐Maqueda et al., 2012), to limit the variation in results between samples. Specific primers for human MUC2, MUC3, MUC13, and MUC17 (PrimePCR^™^ Template for SYBR^®^ Green Assay, human) were purchased from Bio‐Rad (Hercules, CA, USA). Real‐time RT‐PCR (20 μl) was carried out with 10 ng of cDNA, 250 nM primers, and 1× Fast SYBR Green Master Mix (Applied Biosystems, Foster City, Canada) following the manufacturer's instructions.

PCR was carried out in a StepOnePlus real‐time PCR System (Applied Biosystems) by using the recommended thermal cycling conditions (hold 95°C, 10 s; 40 cycles: 95°C, 15 s; 60°C, 60 s; melt curve stage: 95°C, 15 s; 60°C, 60 s). Relative gene expression values were calculated by using β‐actin as the endogenous reference gene and cells with pure DMEM as the calibrator samples (control samples). The amplification of target genes and the endogenous reference gene was analyzed using three biological replicates, each with three technical replicates. The relative abundance of transcripts was calculated as per the report by Livak and Schmittgen (2001). Statistical analysis (treated vs. control) was performed by the nonparametric Wilcoxon signed‐rank test by using GraphPad InStat v. 6.05 (GraphPad Software, San Diego, CA, USA).

### 2.5 ELISA test

After 48 hr of treatment, secretion levels of MUC2 were measured using the Human MUC2 kit from Antibody Research (St. Charles, USA) as per the manufacturer's instructions. The contents in wells were concentrated in a Scanvac centrifugal concentrator (Labogene, Denmark) to 20% of the original volume. The ELISA plate was read at 450 nm on the Tecan Infinite reader. The mucin profile of each sample was evaluated in duplicate, and the values were expressed as ng/ml of proteins in the cell supernatant.

### 2.6 Statistical analysis

Statistical analysis was performed using the statistics software GraphPad Prism version 6.0 (GraphPad Software, USA). All experiments were performed in a minimum of three replicates; results are represented as the mean ± standard deviation (*SD*) and standard error of measurement (*SEM*). Differences were considered significant when *p *<* *0.05.

## 3 RESULTS

### 3.1 Toxicity of polyphenols in the intestinal model

**Figure 1:**
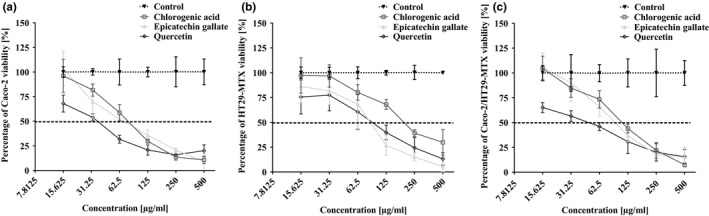
Effect of chlorogenic acid, epicatechin gallate, and quercetin on the viability of Caco‐2 (a), HT29‐MTX (b), and cocultured Caco‐2/HT29‐MTX (c) cells determined by the MTT assay. Values are expressed as means of the percentage of cell viability with respect to control cells ± standard deviation (*SD*), *N* = 3

The MTT assay, which measures mitochondrial activity in viable cells, was evaluated in the Caco‐2/HT29‐MTX coculture and Caco‐2 and HT29‐MTX cultures after 72 hr treatment with chlorogenic acid, epicatechin gallate, and quercetin. A twofold dilution series of 1,280, 640, 320, 160, 80, and 40 μmol/ml were used for the analysis. The percentage of mortality for each concentration was plotted and fit with a sigmoidal curve in order to determine the 50% growth inhibitory concentration (IC_50_). The MTT assay was also carried out for the individual cell lines, in order to establish that the decrease of cell viability was not because of coculture. All the polyphenols studied inhibited the viability of all the intestinal cell lines in a dose‐dependent manner. Caco‐2 cells were more sensitive to all the polyphenols (Figure 1a) than HT29‐MTX cells (Figure 1b) and were inhibited by concentrations of quercetin exceeding 80 μmol/ml or even lower. After treatment with MTT for 2 hr, viability of cells in the coculture of Caco‐2/HT29‐MTX decreased in the presence of up to 167 μM of all the polyphenols (Figure 1c). Therefore, the concentration of polyphenols used did not exceed 10 μM in subsequent experiments, which is still considered as a physiological dose achievable with a normal diet. However, this concentration did trigger slight toxicity. The toxicity results against the coculture of Caco‐2/HT29‐MTX, expressed as IC_50_values, were 337.4, 204.5, and 167.7 μM for chlorogenic acid, epicatechin gallate, and quercetin, respectively. Chlorogenic acid was the least toxic compared to the other compounds, whereas quercetin, at the lowest tested concentration, modestly affected cell viability by 42%.

### 3.2 Mucin gene expression as modulated by the polyphenols

**Figure 2:**
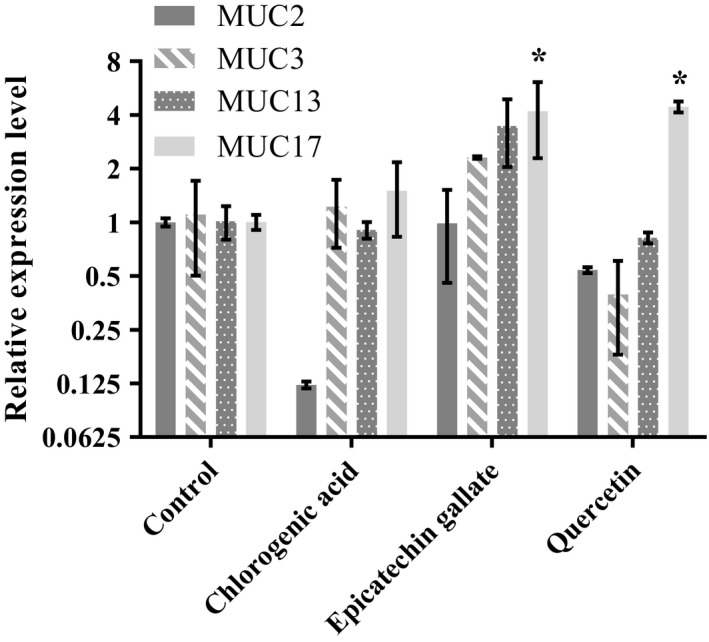
Comparison of the effects of various polyphenols (10 μM) on the expression of various mucins. Values are means of three independent replicates ±*SEM*. Asterisk (*) indicates significance of *p *<* *0.05 by nonparametric Wilcoxon signed‐rank test

Real‐time RT‐PCR was performed to determine the effects of chlorogenic acid, epicatechin gallate, and quercetin on the transcription levels of MUC2, MUC3, MUC13, and MUC17 in the Caco‐2/HT29‐MTX coculture. The cells were exposed to sub‐lethal concentrations (10 μM) of each of the different polyphenol compounds over a period of 48 hr. The results showed that each compound exerts differential control over the expression patterns of mucin genes (Figure 2). MUC17 levels were mostly upregulated in the coculture upon treatment with all of the polyphenols; in particular, quercetin and epicatechin gallate demonstrated statistically significant increases (*p *<* *0.05) in MUC17 levels. Epicatechin gallate and chlorogenic acid resulted in upregulation of MUC13 levels but also led to elevated expression of other membrane‐bound mucins. MUC2 levels, on the other hand, were downregulated after treatment with either chlorogenic acid or quercetin. In the case of epicatechin gallate, the MUC2 expression remained more or less constant. MUC3, MUC13, and MUC17 levels were permanently elevated upon treatment with chlorogenic acid. Thus, expression of MUC17 was elevated after all treatments; however, the other mucins showed compound‐specific patterns.

### 3.3 ELISA assay of polyphenols

**Figure 3:**
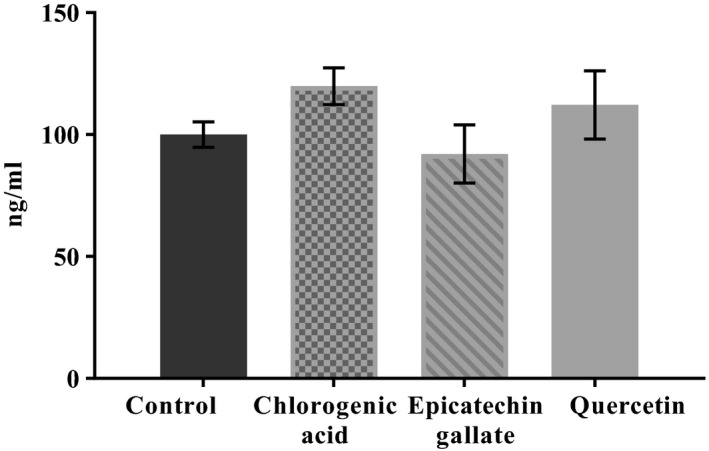
MUC2 protein level in coculture of Caco‐2/HT29‐MTX cells treated with 10 μM of chlorogenic acid, epicatechin gallate, or quercetin determined by ELISA. Values are expressed as means ± *SD* of three independent replicates

To evaluate whether the modulation of mucin mRNA abundance translated to altered mucin protein abundance, the relative level of MUC2 protein was measured by indirect ELISA after 48 hr of treatment with the individual polyphenol compounds (Figure 3). No significant differences in the levels of secreted mucins were found.

## 4 DISCUSSION

Polyphenols are ingested by humans as complex mixtures immersed in a food matrix as part of the normal diet. Approximately 90–95% of dietary polyphenols are not absorbed by the small intestine and therefore reach the colon (Clifford, 2004). We chose to study three phenolic compounds that are ordinarily present in the human diet. Each of the polyphenolic components was examined at a concentration of 10 μM, since this concentration is close to the amount that is expected to be present in the gut and involved in polyphenol–gut interactions as per our toxicity measurements (Deprez, Mila, Huneau, Tome, & Scalbert, 2001; Peng & Kuo, 2003). Our data are also in good agreement with the data reported by O'Hara et al. (2006) published recently, which showed that the cells may induce mucin expression.

Epithelial cells lining the gut are regularly exposed to digested mixtures of polyphenols. The chemical structures and the antioxidant potential of individual phenolic compounds can activate genes encoding different mucins. To the best of our knowledge, only a few studies on the association between mucus production and polyphenol ingestion have been published to date (Hervert‐Hernandez & Goñi, 2011; Lee et al., 2010). The current study focuses on the change in expression of single mucins (MUC2, MUC3, MUC13, and MUC17) induced by individual polyphenols: chlorogenic acid, epicatechin gallate, and quercetin. Some dietary compounds may cause the production of different end products from bacterial metabolism and thus can modify the expression of mucin genes and proteins leading to an increase in mucus layer thickness (Duda‐Chodak, 2012; Kim et al., 2008). As mentioned above, in the present study, we found that each phenolic compound induces differential expression patterns of mucin genes. The effect of polyphenols on mucin barrier might be complex as previous work indicated that polyphenols from tea (epigallocatechin gallate and epicatechin) act as cross‐linkers of intestinal mucins (Georgiades, Pudney, Rogers, Thornton, & Waigh, 2014). Polyphenols, such as epigallocatechin gallate and epicatechin, may exert beneficial effects on human health, not only as simple antioxidants acting as free‐radical scavengers, but also by indirectly interfering with specific signaling proteins, which mediate gene regulation in response to oxidative stress and inflammation (Biasi et al., 2013). In the study by Kim et al. (2008), it was found that (−)‐epigallocatechin‐3‐gallate markedly suppressed IL‐1β‐induced MUC5AC gene expression and secretion. Our analysis, in accordance with previously published results, showed that dietary polyphenols downregulated MUC2 in Caco‐2/HT29‐MTX cocultured cells and may possibly alter its biosynthesis or secretion, which may be linked to gastrointestinal disease (Rakha et al., 2005; Van Klinken, Van der Wal, Einerhand, Büller, & Dekker, 1999). Further, in partial agreement with the study by Lugli et al. (2007), MUC2 down‐regulation is a marker of tumor progression through the adenoma–carcinoma sequence and survival of colorectal carcinoma. We also obtained evidence that chlorogenic acid and epicatechin gallate increased the level of MUC3. In accordance with our findings, a study by Rakha et al. (2005) demonstrated that MUC3 increases during cancer development; however, this mucin is generally expressed at low levels in the colon. Similarly, the membrane‐bound MUC13 was upregulated by epicatechin gallate, while quercetin and chlorogenic acid did not cause a significant change in its expression levels. Furthermore, the study by Yang, Lee, Chen, and Yang (1997), based on animal models, confirmed that the catechins are able to inhibit the formation of tumors in different organs. This study showed that epicatechin, and thus possibly the other polyphenols, can reduce cancer cell proliferation. It should also be noted that MUC13 is a good differentiation marker for gastrointestinal mucosa and that it may have a causal role that correlates with two distinct gastric tumorigenesis pathways. The aforementioned findings are in agreement with the literature available on this topic (Shimamura et al., 2005). Our results also indicate that the polyphenols used in this study upregulated the expression of MUC17, which is consistent with a previous finding that the expression of MUC17 increases in the presence of polyphenols (Senapati et al., 2010). Our results, in accordance with prior investigations, confirm that the examined polyphenols play an important role in regulating mucin expression, which may also be related to cancer development.

## 5 CONCLUSION

In conclusion, our study demonstrated that polyphenols modulate mucin expression, which may affect bacteria that utilize mucin to protect the digestive tract from pathogens, and may be linked to cancer progression, although a causal link has not yet been established. The use of micronutrients found in certain foods appears to aid in the prevention and treatment of gastrointestinal tract diseases. Further research is needed to determine the specific role of these particular mucins in relation to the beneficial effects of the micronutrients.

## ETHICAL STATEMENT

I testify on behalf of all coauthors that this article is original and has not been published and will not be submitted for publication elsewhere. The manuscript does not contain experiments using animals or human.

## CONFLICT OF INTEREST

The authors have declared no conflict of interest.
